# Time to rethink circadian rhythms beyond the transcriptome

**DOI:** 10.1093/jb/mvag031

**Published:** 2026-07-01

**Authors:** Yuta Otobe, Hikari Yoshitane

**Affiliations:** Circadiain Clock Project, Tokyo Metropolitan Institute of Medical Science, Kamikitazawa 2-1-6, Setagaya-ku, Tokyo 156-8506, Japan; Circadiain Clock Project, Tokyo Metropolitan Institute of Medical Science, Kamikitazawa 2-1-6, Setagaya-ku, Tokyo 156-8506, Japan; Graduate School of Science, The University of Tokyo, 7-3-1 Hongo, Bunkyo-ku, Tokyo 113-0033, Japan

**Keywords:** circadian rhythm, proteomics, mass spectrometry, post-translational modification, protein localization

## Abstract

The circadian clock generates ~24-hour rhythms in physiology by coordinating gene expression programs, but temporal mRNA profiles often fail to predict their protein function rhythms. This gap reflects regulatory layers beyond transcription, including rhythmic translation, protein stability, subcellular localization and post-translational modifications that collectively determine the circadian rhythms of protein abundance and activity. Here, we summarize evidence supporting a shift from RNA-level descriptions to protein-level frameworks and readouts of the circadian rhythms. Here, we highlight three topics: (i) protein abundance rhythms as informative but incomplete readouts, (ii) widespread circadian control of nuclear localization and phosphorylation that can occur without changes in total protein levels, and (iii) multi-tissue proteomic comparisons that reveal how circadian rhythms are organized differently across tissues. We then discuss how recent data-independent acquisition-based, high-throughput mass spectrometry accelerates cross-study reuse and hypothesis generation, as illustrated by a mouse circadian proteome atlas and an interactive portal enabled by Orbitrap Astral mass spectrometer. Together, these advances motivate ‘functional chronobiology’, linking proteome dynamics to mechanism and disease-relevant physiology.

## Graphical Abstract

**Graphical Abstract:**
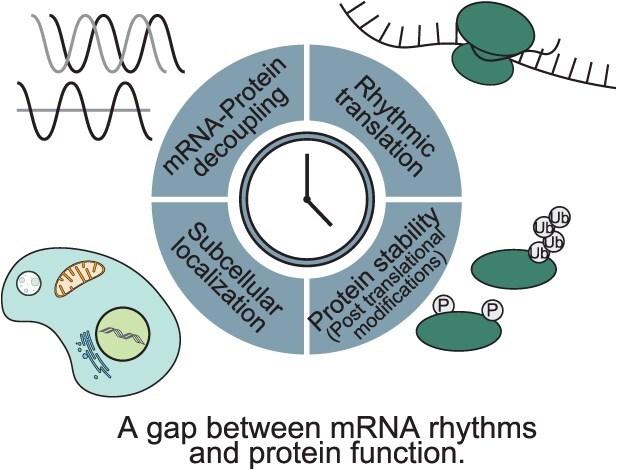

The circadian clock generates ~24-hour rhythms in physiology and behaviour by orchestrating time-dependent gene expression. Over the past two decades, large-scale transcriptomic studies have revealed that approximately 10% to 20% of expressed genes exhibit circadian rhythms in any given tissue, establishing rhythmic transcription as a central paradigm in circadian biology *(**1–3**)*. These studies have provided a foundational framework for understanding how the clock regulates metabolism, endocrine function and behaviour. Recent large-scale circadian omics resources, including primate multi-tissue diurnal transcriptome atlases and databases that translate rhythmic gene expression into biomedical contexts, have broadened the impact of chronobiology across comparative and translational research *(**4–6**)*.

However, cellular function is governed by proteins rather than mRNAs. Increasing evidence indicates that rhythmic mRNA expression does not necessarily translate into rhythmic protein abundance or activity, owing to delays in translation, protein stability, subcellular localization and post-translational modifications (PTMs) *(**7–12**)*. This conceptual gap can be described as a decoupling of circadian rhythmicity across molecular layers: mRNA abundance, total protein abundance and protein state variables, such as PTMs or subcellular localization (Fig. 1). As a result, a transcriptome-based information may be insufficient to capture the full landscape of the circadian regulation. Recent advances in mass spectrometry-based proteomics now allow systematic interrogation of protein abundance, subcellular localization and PTMs with unprecedented sensitivity and quantitative accuracy in protein identification. These developments motivate a conceptual shift from RNA-level circadian regulation toward a protein-level and function-oriented understanding of circadian physiologies.

**Fig. 1:**
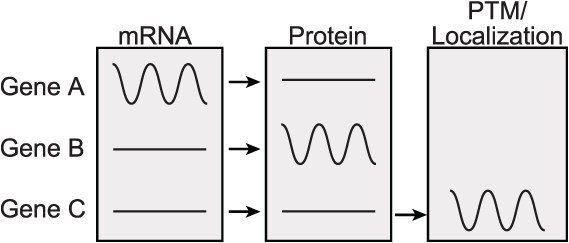
Decoupling of circadian rhythms across molecular layers. Schematic showing that rhythmicity can arise at different regulatory levels: mRNA abundance, total protein abundance and protein activity/state via PTMs or subcellular localization. Genes A to C represent distinct example genes, and arrows indicate layer-to-layer relationships for each gene. Wavy lines indicate rhythmic oscillation, whereas straight lines indicate constant levels (*e.g.* Gene C shows PTM/localization rhythms despite constant mRNA/protein).

In this review, we summarize why transcriptome data are insufficient to understand circadian rhythms, then organize emerging evidence for protein-level regulation into two layers: (i) protein abundance rhythms and (ii) subcellular localization and PTM rhythms. We then discuss how recent technological advances, especially data-independent acquisition (DIA) workflows and high-throughput instruments, enable next-generation circadian proteome atlases, using the 2,026 mouse Circadian Proteome Atlas as a case study *(**13**)*.

## Transcriptome-Driven Circadian Biology and Technical Limitation

Genome-wide expression profiling transformed chronobiology by revealing the pervasive influence of the clock on transcription *(**14**)*. Early microarray and RNA-seq studies demonstrated coordinated rhythmic transcription across metabolic and signalling pathways in multiple tissues *(**1**,*  *15**)*. Subsequent multi-tissue atlases further highlighted tissue-specific rhythmic expression and their relevance to physiology and medicine *(**3**)*. Despite these successes, several fundamental limitations have become apparent. First, the phase of protein abundance often lags behind that of its corresponding mRNA by several hours, reflecting the time required for translation and protein maturation *(**7**)*. Second, many rhythmic transcripts do not produce detectable protein rhythms, whereas numerous rhythmic proteins arise from non-rhythmic mRNAs *(**7**,*  *16**)*. Third, although transcriptome- and chromatin-centered approaches have greatly refined the transcriptional architecture of circadian regulations, they do not capture protein state variables, such as PTMs, protein interaction or subcellular localization, which ultimately govern protein function *(**17–20**)*.

These discrepancies motivate a framework in which transcription is an important input layer, but protein-level regulation is required to understand circadian physiologies.

## Circadian Biology Revealed by the Proteomics

Early circadian proteomic studies revealed that a substantial fraction of proteins oscillate in abundance, often with phases distinct from their corresponding mRNAs *(**7**,*  *16**)*. These findings underscored the importance of post-transcriptional regulation in shaping circadian rhythms. However, technical challenges limited most early analyses to single tissues and modest proteome depth, leading to poor detection and variable quantification of low-abundance factors, such as transcription factors and signalling molecules. Beyond detection limits, mRNA–protein phase discordance is not limited to low-abundance proteins, supporting a broad role of post-transcriptional control in shaping circadian protein outputs *(**7**,*  *8**)*. In particular, circadian regulation of translational efficiency can modulate protein production independently of mRNA rhythms, providing a direct mechanism for mRNA–protein phase uncoupling *(**10**,*  *11**)*. Yet protein abundance alone remains insufficient to capture functional dynamics, because protein activity is frequently governed by subcellular localization and PTMs rather than quantity *(**21**)*. Previous high-depth nuclear proteomics shows that many rhythmic nuclear proteins lack corresponding whole-cell abundance rhythms, consistent with circadian gating of nuclear trafficking and compartment-specific proteostasis beyond synthesis or degradation *(**19**,*  *22**)*. In parallel, phosphoproteomics demonstrates that rhythmic phosphorylation is widespread; for example, in mouse liver more than 20,000 phosphosites were quantified and ~ 25% showed significant circadian oscillations *(**20**)*, and that phosphosite dynamics can encode time-of-day–specific signalling states via circadian regulation in inferred kinase activities *(**20**,*  *23**,*  *24**)*. Multisite phosphorylation of core clock proteins exemplifies how phosphorylation regulates stability, localization and transcriptional repression *(**25**,*  *26**)*, and similar principles extend to pathways in metabolism, neuronal signalling, and proteostasis. Consistent with this view, deep multi-tissue proteomic atlases indicate that abundance rhythms are widespread but represent only one component of circadian regulation *(**19**,*  *22**)*.

**Fig. 2:**
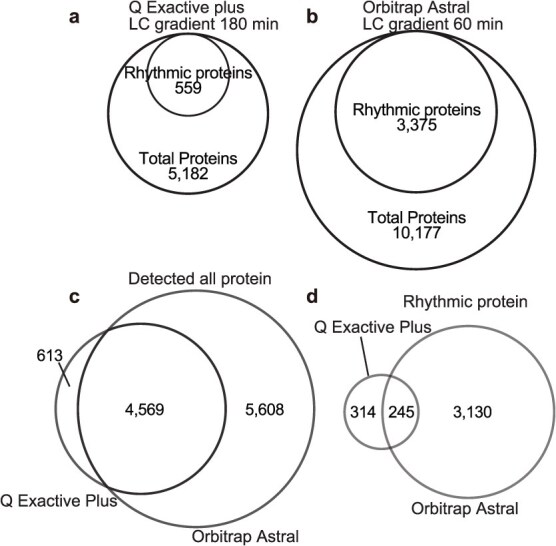
Rhythmic protein detection in mouse liver nuclear fractions across the circadian cycle. Liver nuclei were collected at CT2, CT6, CT10, CT14, CT18 and CT22, and 1 μg of peptides was injected per run. Samples were analysed by DIA on (A) a Q Exactive Plus with a 180-minute LC gradient or (B) an Orbitrap Astral with a 60-minute LC gradient. Proteins were identified/quantified with DIA-NN, and rhythmic proteins were called by BioCycle; total and rhythmic protein numbers are indicated. (C and D) Overlap of all detected proteins (C) and rhythmic proteins (D) in the Q Exactive Plus and Orbitrap Astral datasets.

## Proteomics Technology Innovations and the Otobe et al. Case Study

**Fig. 3:**
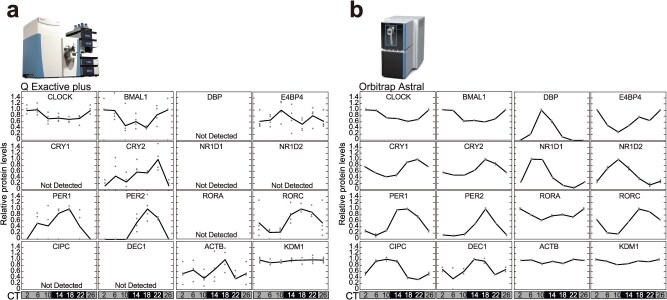
Improved detection of core clock proteins in liver nuclear proteomes using Orbitrap Astral. Mouse liver nuclear fractions were collected at CT2, CT6, CT10, CT14, CT18 and CT22 and analysed by DIA-MS on (A) a Q Exactive Plus or (B) an Orbitrap Astral. Relative protein levels for representative clock proteins are shown across circadian time; grey dots indicate individual measurements and black lines denote the mean at each time point. ‘Not detected’ indicates proteins not quantified in the corresponding dataset. ACTB and KDM1 are shown as non-rhythmic reference proteins. CT: circadian time under constant darkness, with CT0 and CT12 defined as the onsets of subjective day and subjective night.

One of the major barriers in circadian proteomics has been the need to measure many samples (driven by multiple time points, tissues, subcellular fractions and phospho-peptide enrichment), while preserving sufficient proteome coverage and quantitative consistency. DIA strategies, supported by computational advances, improved reproducibility across large cohorts and made short-gradient proteomics practical for time-series designs *(**27–30**)*. Next-generation platforms further eased the long-standing trade-off between proteome coverage and throughput. The Orbitrap Astral mass spectrometer, for example, combines high resolving power and mass accuracy with MS/MS scan speeds up to ~200 Hz, enabling both rapid measurement and deep proteome coverage in high-throughput settings *(**31–33**)*. In addition to improvements in quantitative consistency and throughput driven by DIA and high scan speed, the Orbitrap Astral architecture is also designed to enhance ion transmission and detection efficiency, which can improve practical sensitivity and enable identification of lower-abundance proteins in complex samples. A direct comparison highlights the impact of these advances on circadian proteomics. Using identical liver nuclear circadian samples, DIA analysis on a Q Exactive Plus, a former mass spectrometer, with a 180-minute gradient quantified 5,182 proteins (559 rhythmic), whereas an Orbitrap Astral with a 60-minute gradient quantified 10,177 proteins (3,375 rhythmic), highlighting a substantial gain in depth and rhythmic protein discovery despite a threefold shorter LC gradient (Fig. 2A and B). We additionally assessed cross-platform overlap and found that the Orbitrap Astral recapitulated most proteins quantified on Q Exactive Plus (4,569 shared proteins; ~88% of the Q Exactive Plus set), while substantially expanding coverage and rhythmic protein discovery (Fig. 2C and D). Notably, Orbitrap Astral markedly improved detection of core clock proteins recovering multiple low-abundance factors that were not quantified on Q Exactive Plus under these conditions (Fig. 3). Together, these gains make it feasible to pair dense sampling with fractionation schemes such as nuclear extracts to recover low-abundance factors that remain challenging even on advanced instruments *(**13**)*.

As a case study, Otobe et al. *(**13**)* built a mouse Circadian Proteome Atlas by profiling a broad panel of 32 tissues under a unified experimental and analytical framework, including the SCN and multiple brain regions, with matched whole-cell and subcellular fraction measurements where appropriate, and further extending the design to additional context datasets, including developmental and disease-related samples using Orbitrap Astral-based workflows. Across 584 samples, the study quantified 18,956 proteins, enabling standardized, cross-tissue comparability at scale, an important prerequisite for distinguishing ‘ubiquitous clock outputs’ from tissue-enriched programs and for defining region-enriched markers within the brain *(**13**)*.

The same study further shows how combining subcellular fractionation with phosphoproteomics can reveal protein regulation beyond abundance that are largely invisible to RNA profiles: (i) extensive nuclear remodelling in which many proteins show rhythmic nuclear behaviour despite weak or absent whole-cell rhythms, and (ii) widespread rhythmic phosphorylation that can occur even when total protein abundance remains essentially constant *(**13**)*. Together, these results help reframe circadian rhythms as ‘when, where and which proteins act’, rather than only ‘which RNAs oscillate’.

**Fig. 4:**
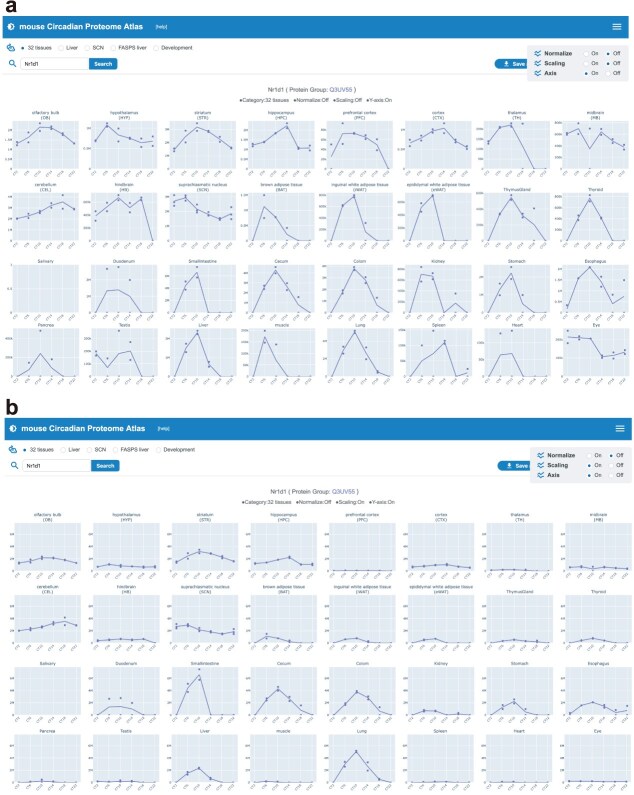
Using the Mouse Circadian Proteome Atlas web portal. To query protein profiles across tissues, select ‘32 tissues’ and enter a gene/protein name in the search box. The portal displays circadian time plots for the selected protein across tissues. The *y*-axis shows DIA-NN protein intensity values (arbitrary units), which represent relative intensity estimates rather than absolute abundance or copy number. (A) With Scaling OFF, each tissue trace is rescaled so that its maximum mean value is set to 1, enabling comparison of temporal patterns (phase and waveform) across tissues. (B) With Scaling ON, raw quantitative values are shown, allowing comparison of relative protein abundance between tissues within this dataset under the same LC–MS conditions and a unified analysis pipeline; these values should not be interpreted as absolute molar quantities and may still be influenced by tissue-specific matrix effects and peptide detectability.

To facilitate reuse of these datasets, we also provide an interactive web portal (https://chronoproteinology.org/circadian_atlas) that enables gene-centric exploration of circadian protein and phospho-peptide profiles across tissues and datasets, with options to export the displayed results (Fig. 4). Such a portal lowers the barrier from atlas-scale measurement to hypothesis-driven experiments, for example, by allowing rapid cross-tissue comparisons of phase and amplitude, and by making candidate regulators immediately testable in targeted follow-up studies. This type of resource-oriented dissemination is increasingly important for converting large-scale circadian proteomics into testable hypotheses and community-wide discovery.

## Perspective: Toward Functional Chronobiology

Collectively, emerging proteomic data support a hierarchical model of circadian regulation encompassing transcription, translation, subcellular localization and PTMs. In this framework, rhythmic mRNA expression often represents an upstream layer, while protein-level regulation defines functional outputs.

This protein-centered view has broad implications. It provides mechanistic hypotheses for circadian misalignment and clock-related disorders that may arise from disrupted phosphorylation or mis-timed compartmentalization rather than transcription alone. It also creates new opportunities for chronopharmacology, because drug targets and metabolic enzymes can exhibit time-of-day-dependent PTMs or compartment-specific regulation that influence efficacy and toxicity *(**34**,*  *35**)*. Looking forward, the key challenge is to move from description to causality by integrating proteomics with targeted perturbations, quantitative signalling models and functional assays that directly link protein-level rhythms to physiological outcomes. Next-generation atlases and accessible portals will accelerate this transition by enabling hypothesis generation across tissues and contexts. Looking ahead, building on established resources, such as CircaDB and CircadiOmics, next-generation atlases and clinically oriented portals should further accelerate cross-study reuse and hypothesis generation *(**5**,*  *36**,*  *37**)*.

As circadian biology enters this era of routine deep proteomics, functional chronobiology, anchored in when, where and which proteins act over time, promises a more mechanistic understanding of biological timekeeping.
